# Magnesium lactate in the treatment of Gitelman syndrome: patient-reported outcomes

**DOI:** 10.1093/ndt/gfw019

**Published:** 2016-03-03

**Authors:** Caroline M. Robinson, Fiona E. Karet Frankl

**Affiliations:** Department of Medical Genetics and Division of Renal Medicine, University of Cambridge and Cambridge University Hospitals NHS Trust, Cambridge, UK

**Keywords:** Gitelman syndrome, hypomagnesaemia, patient-reported outcomes, tubulopathy

## Abstract

**Background:** Gitelman syndrome (GS) is a rare recessively inherited renal tubulopathy associated with renal potassium (K) and magnesium (Mg) loss. It requires lifelong K and Mg supplementation at high doses that are at best unpalatable and at worst, intolerable. In particular, gastrointestinal side effects often limit full therapeutic usage.

**Methods:** We report here the analysis of a cohort of 28 adult patients with genetically proven GS who attend our specialist tubular disorders clinic, in whom we initiated the use of a modified-release Mg preparation (slow-release Mg lactate) and who were surveyed by questionnaire.

**Results:** Twenty-five patients (89%) preferred the new treatment regimen. Of these 25, 17 (68%) regarded their symptom burden as improved and seven reported no worsening. Of the 25 who were not Mg-treatment naïve, 13 (59%) patients reported fewer side effects, 7 (32%) described them as the same and only 2 (9%) considered side effects to be worse. Five were able to increase their dose without ill-effect. Overall, biochemistry improved in 91% of the 23 patients switched from therapy with other preparations who chose to continue the modified-release Mg preparation. Eleven (48%) improved both their Mg and K mean levels, 3 (13%) improved Mg levels only and in 7 cases (30%), K levels alone rose.

**Conclusions:** Patient-reported and biochemical outcomes using modified-release Mg supplements were very favourable, and patient choice should play a large part in choosing Mg supplements with GS patients.

## INTRODUCTION

Gitelman syndrome (GS) is a rare inherited renal electrolyte wasting disorder primarily characterized by hypokalaemia, hypochloraemic metabolic alkalosis, hypomagnesaemia and hypocalciuria. The condition was first described in 1966 [1]. It was originally thought to be a variant of Bartter syndrome until 1996 when Simon *et al.* [2] identified the underlying causative mechanism as loss-of-function mutations in *SLC12A3*, which encodes the sodium chloride co-transporter in the distal convoluted tubule. Inheritance is autosomal recessive and prevalence is estimated at 1:40 000 (http://ghr.nlm.nih.gov/condition/gitelman-syndrome).

GS symptoms are predominantly related to chronic hypokalaemia and hypomagnesaemia and commonly include generalized fatigue, muscle weakness, muscle cramps, thirst, polyuria, carpopedal spasm, paraesthesiae, palpitations and joint pain. More severe manifestations such as chondrocalcinosis requiring joint replacement [3], seizures, rhabdomyolysis, cardiac arrhythmias and sudden cardiac arrest have also been reported [4]. The frequency and severity of symptoms and signs are variable, but contrary to earlier descriptions of GS, and as reported by Cruz *et al.* in 2001 [5], few patients are truly asymptomatic.

The aims of treatment are to improve patient symptoms, quality of life and serum electrolyte levels, and to ensure cardiac rhythm stability. However, it is often not possible to achieve electrolyte levels within the normal range because of poor tolerability of available medications. Standard treatment includes the use of a high-salt/potassium/magnesium diet and oral magnesium (Mg) and potassium (K) supplements, sometimes together with K-sparing diuretics (concomitant hypotension permitting). The need for Mg and K replacement is lifelong and oral dose requirements may be very high. Intravenous electrolyte replacement is best reserved for critically low serum Mg/K, emergency situations or elective surgery in GS patients.

While hypomagnesaemia is a common finding in the chronically sick, the elderly, in malabsorbtive syndromes and in dietary deficiency, very little information exists concerning the bioavailability, efficacy and tolerability of Mg compounds in humans. Studies have largely been limited to healthy volunteers or animal models [6–9]. This may be because Mg is classed as a nutritional supplement rather than medication [10] and also perhaps because hypomagnesaemia, and its sequelae, are under-recognized in clinical practice [11, 12].

Of a number of reports of Mg use in heart disease, asthma or pre-eclampsia [13–15], none addressed either side effects or Mg formulation. Only one compound comparison study has been published in patients with chronic gastrointestinal (GI) disorders [16]; there are no studies of those with renal electrolyte loss. The choice of Mg supplementation in clinical practice is therefore usually decided on factors such as cost, compound availability, and physician and/or patient preference.

High doses of Mg are often required to combat the excessive urinary losses found in GS. However, oral Mg preparations are frequently poorly tolerated due to their laxative and other GI effects. A careful balance is required in order adequately to replace renal Mg loss without inducing counter-productive GI Mg loss and this can present a therapeutic challenge. In an effort to improve efficacy and quality of life in these patients, we trialled the use of slow-release Mg lactate (SRMgL) in a cohort with genetically proven GS and evaluated their experiences. SRMgL is designed for twice-daily administration rather than the thrice-(or more) daily requirement of other preparations.

## MATERIALS AND METHODS

We approached patients with genetically proven GS currently under care in our specialist nephrology clinic. All had previously provided ethically approved written consent for research (NRES 08/H0306/62) and a purpose-designed questionnaire was sent to them after a period of not <4 months since commencing SRMgL. As well as collecting demographic, dose/length of treatment and previous Mg treatment data, patients were asked to report side effects, impact on GS symptoms, residual GS symptoms and subjective opinion of impact on their biochemical results. In addition, where Mg supplements had previously been used, patients were invited to compare their prior regimen with SRMgL in respect of side effects, number of tablets taken per day and ease of swallowing.

Based on the manufacturer's claims for bioavailability, each subject's initial dose of SRMgL was calculated at two-thirds the tablet number of their previous Mg formula, or in treatment-naïve individuals, one tablet bd. Doses were titrated upward to maximum tolerability and/or serum Mg >0.6 mmol/L (1.2 mEq) where possible; changing preparations to maximize tolerability is a routine part of our clinical regimen. Questionnaire responses were evaluated by quantitative and qualitative data analysis.

We also retrospectively analysed serial biochemical data for those patients for whom at least two pre- and four post-commencement SRMgL values were available. The Mann–Whitney *U*-test was used to compare mean pre-treatment and post-treatment values, with statistical significance determined by P ≤ 0.05, and Fisher's exact test to compare proportions of patients reaching target treatment values pre- and post-SRMgL.

## RESULTS

From 37 invitations to participate, 28 questionnaires were returned (76%) by 21 female and 7 male patients (3:1), with median age 39 years (range 20–74 years). Twenty patients (71%) had formerly received treatment with maximally tolerable doses of Mg glycerophosphate prior to switching to SRMgL. An additional five patients had variously been prescribed Mg oxide, Mg chloride, Mg aspartate or Mg glycinate prior to attendance at our clinic, and three had not previously been treated with Mg supplements. Prior to switching, the median daily dose of Mg supplement in the 22 out of 25 for whom data were available was 7 tablets (range 4–32; 32–256 mEq Mg).

Given the difficulties of treating patients with high-dose Mg compounds, patient responses were very positive regarding SRMgL, with almost 90% (*n* = 25) choosing to continue long term, and only 3 out of 28 discontinuing this medication in favour of their previous preparation. Two of these three discontinued SRMgL within 48 h due to difficulty swallowing the tablets, which are caplets 2 cm in length. The third patient, experiencing the same difficulty, broke the tablets in half (the manufacturer's information suggests this is acceptable; consequently, the tablets are scored). However, this individual experienced increasing stomach pain and had to discontinue treatment after 3 weeks. Her gastric symptoms then resolved spontaneously, although it is worth noting she had a prior history of gastro-oesophageal reflux disease requiring gastric fundoplication. A further three patients disliked the size of the tablets but were able to continue. All six of these subjects had previously received Mg therapy with a different preparation. SRMgL doses in those continuing ranged from 2 to 18 tablets daily (range 14–126 mEq Mg)/day with a median of 6 tablets (42 mEq/day).

The overall rating of side effects in comparison with previous Mg was favourable, with 13 (59%) patients reporting fewer side effects, 7 (32%) describing them as the same and only 2 (9%) considering side effects to be worse (one tolerated a minor increase in laxative effect as they were able to reduce their total number of tablets, one required extra Mg supplementation when she became pregnant but was unable to tolerate more than 10 tablets/day—she was able to successfully take a combination of SRMgL and Mg glycerophosphate).

In common with all other Mg preparations, the laxative effect of SRMgL was the most frequently described side effect among the cohort (Table 1). Unexpectedly, however, eight patients (32%) experienced no GI effects at all, with an additional four (16%) commenting on only very mild loosening of stool. Six subjects (25%) reported a moderate laxative effect (accompanied by mild stomach cramps in two cases). Of the three patients (12.5%) suffering more severe GI upset, dose reduction was sufficient to improve tolerability in all, with all participants eventually tolerating 2–12 tablets/day. The median dosage time was 19 months (range 4–40). In the treatment-naïve subgroup, one subject reported no GI side effects, one experienced moderate laxative effect with stomach cramps and one commented on increased thirst only.

**Table 1:** Reported side effects in 25 patients continuing SRMgL

Reported side effects	No. of pts	%
Difficulty swallowing tablets	3	12
None	8	32
Laxative effect: mild	4	16
Laxative effect: moderate	6	24
Laxative effect: severe/dose limiting	3	12
Abdominal cramps associated with laxative effect	2	8
Increased thirst	1	4
Insomnia	1	4

Three patients were able to increase their SRMgL dose when they experienced fewer side effects than with their previous preparation, while two were able to increase SRMgL with a similar side-effect profile. Two patients decided to take SRMgL in combination with glycerophosphate as they said it benefited them best.

Patients were asked to evaluate the severity of their GS symptoms on SRMgL. In 17 cases (68%), they reported that symptoms had improved ‘a lot’ (*n* = 8) or ‘a little’ (*n* = 9). Seven (28%) experienced no change and only one (4%) felt that his symptoms were a little worse. None of the cohort described significant worsening of their overall clinical condition. In the treatment-naïve subgroup, one subject described a good improvement in symptoms, one reported a small improvement and one felt that symptoms were unchanged.

Notably, and contrary to the early GS literature where the condition was described as largely asymptomatic [17, 18], only six (24%) subjects rated themselves as symptom free, while a further three (12%) described GS symptoms as minor. Thus, 16 (64%) patients continued to have residual GS symptoms.

Nineteen patients (76%), including two who were Mg naïve, subjectively reported improvements in their serum electrolyte levels, 6 (24%) reporting a good improvement and 13 (52%) a modest improvement. In five cases (20%), electrolyte results were thought by patients to be unchanged and one patient (4%) thought blood results were a little worse since commencing SRMgL. None of the group felt that their electrolyte levels had significantly deteriorated.

**FIGURE 1:**
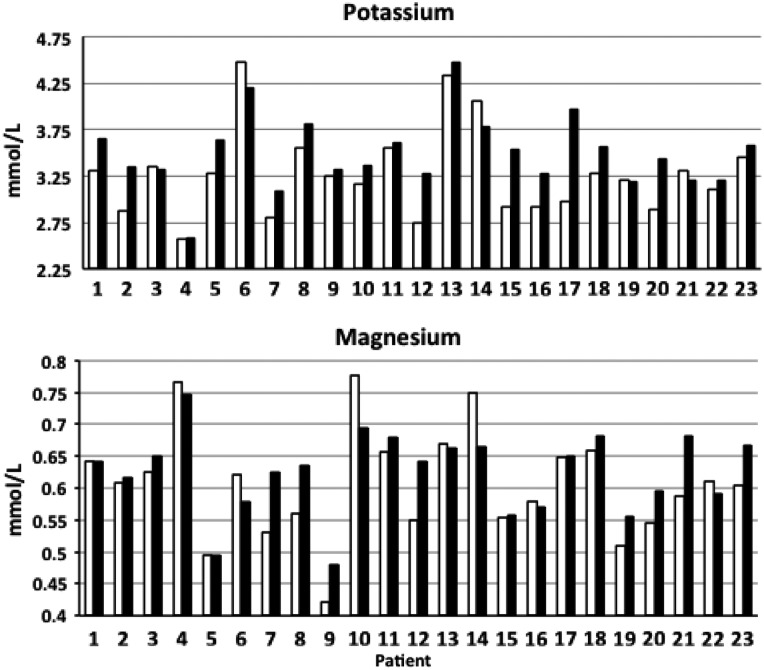
Mean pre-SRMgL (white bars) and post-SRMgL (black bars) potassium and magnesium values for the 23 patients who continued SRMgL.

**FIGURE 2:**
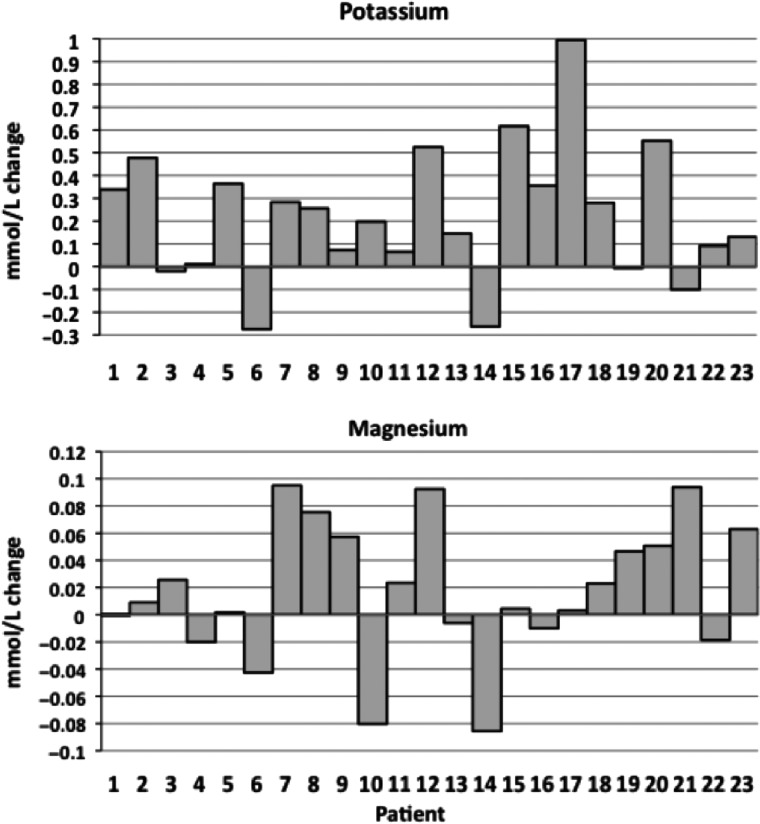
Changes in pre-to-post mean potassium and magnesium levels for the 23 patients who continued SRMgL.

To evaluate these reports more objectively, we examined serum Mg and K values for the 23 patients with sufficient available results; the number of measurements ranged from 2 to 124 (over up to 10 years pre-SRMgL) and 4–34 (over up to 5 years post-SRMgL). Because blood results can be quite variable in GS, we chose to compare the mean pre- and post-treatment values. Overall, this analysis revealed that improvements were in fact evident in 91% of this group (Figure 1). Eleven patients (48%) improved both their Mg and K mean levels, three (13%) improved Mg levels only and in seven cases (30%), only K levels rose (Figure 2). Two (9%) made no gains in either Mg or K mean levels; however, in both cases, levels were well above our minimum target, and one of these (Figures 1 and 2, Patient 14) had been able to discontinue amiloride. The recorded gains were variable and in some cases, gains in one value were offset by modest falls in the other. We found that the post-treatment improvements in K for the cohort reached statistical significance (P = 0.029), but those for Mg did not (P = 0.238). In 14 of the 23 cases, Mg and K moved in parallel (Figure 2).

We looked at how many of the 23 reached mean minimum treatment target levels of serum Mg ≥0.60 mmol/L (≥1.2 mEq) and serum K ≥3.2 mmol/L before and after treatment with SRMgL. In respect of Mg values, 13 (57%) reached the mean target pre- and 16 (69%) post-treatment (not different). For K values, there was a significant difference (P = 0.024), with 20 patients (87%) reaching target levels post-treatment compared with 13 (57%) prior.

Finally, patients who were not previously Mg naïve (22/25) were asked to compare SRMgL with their prior Mg preparation. Eleven of these (50%) reported being able to reduce the number of Mg tablets consumed per day, and in all of these, Mg attained or remained above our minimum target level. For example, Patient 8 (Supplementary data, Table S1) was able to reduce Mg dosing by one-third, but both K and Mg levels rose. For a further six (27%), the number of tablets taken daily was reported as the same and five (23%) tolerated an increase to support their Mg levels. A few individuals noted that the cost of their medications and the large number of pills they had to take contributed in part to low perception of wellbeing.

## DISCUSSION

We report here the subjective and biochemical effects of switching Mg preparations in the context of the rare renal tubulopathy GS. In the absence of clinical guidelines or other data concerning Mg preparations, we had taken a clinical decision to trial the use of SRMgL after one of our patients reported a beneficial side-effect profile, having purchased the tablets while in the USA. Our early impressions of significant improvement from the patient's perspective are supported by the results of this audit.

Slow-release electrolyte preparations are more likely to provide stable therapeutic serum levels in patients with continuous urinary electrolyte loss. This is borne out by the reported improvement in symptoms. However, symptom burden in GS does not always correlate well with serum Mg values [5]; hence, blood results should be assessed in conjunction with patient-reported outcomes. Our positive results are likely to reflect the benefits of the slow-release preparation rather than of the lactate in the compound, for which there is no evidence of specific benefit, but we cannot state this definitively.

Clinical evaluation both of the degree of hypomagnesaemia and the effectiveness of treatment are usually based on measured serum Mg values, yet there are a number of variables to be taken into consideration in GS. There is poorly understood day-to-day variability of both symptoms and electrolyte levels [19]; individual variation in genotype, absorption of Mg, exercise, fluid intake/output, stress, gender, menstrual cycle and concomitant illness may all influence the blood result [5, 20–23]. Time of day of blood drawing is also of relevance, particularly with the short-acting preparations, with values likely to be lowest before breakfast and highest an hour or two after a dose. Using the mean pre- and post-treatment values in our biochemical analysis was therefore a strategic means of minimizing these potential confounders in a real-world clinical setting.

The importance of achieving adequate Mg levels in maintaining serum K is well recognized [24, 25]. Interestingly, the mean serum K levels post-SRMgL in our patients improved more readily than Mg levels themselves. This may be explained by serum Mg not necessarily reflecting Mg storage, since <1% of total body Mg is present in the serum [26]. Patients who are chronically losing Mg are likely to be total-body Mg-deplete, and replenishment will be accompanied by some redistribution into exchangeable stores [27]. In addition, it is possible that a smoothing of overall Mg levels by using a slow-release compound supports stabilization of, or improvement in, K levels.

A wide variety of Mg preparations is available in different countries, most of which are classed as food supplements. For these, there is no requirement to provide the same scientific evidence to support bioavailability, efficacy and tolerability as applies to medicines, and many health-food store-stocked Mg compounds contain doses too low to be useful in GS. Labelling of these ‘over the counter’ preparations is often not very informative and conforms to limited standards, although more stringent labelling regulations were introduced in the USA in 2014 (www.crnusa.org/pdfs/DS-RegsLabel-0613.pdf). In addition, few Mg supplements are licensed; in the UK, this is limited to Mg aspartate (www.medicinesresources.nhs.uk/GetDocument.aspx?pageId=779639). In UK clinical practice outside nephrology, Mg oxide has been the compound most frequently prescribed, but it has much lower bioavailability than organic formulae [9], and in ours and others' experience, poor tolerability when used in larger doses. Consequently, the choice of Mg supplementation is often decided on physician preference based on clinical experience and factors such as cost and compound availability, rather than scientific evidence.

The management of GS has not changed significantly over the past four decades and there is an ongoing need for better treatment options for this and other renal tubulopathies, and for formal research into the pharmacodynamics of such treatments. Because of the rarity of these conditions, adequately powered controlled trials would be very difficult to achieve, and the market for novel therapies would be small. Re-purposing known drugs would potentially be possible, as demonstrated by Blanchard *et al*. [28], who trialled the use of indomethacin in a cohort of GS patients. However, safety issues including adverse impacts on glomerular filtration rate and GI epithelial integrity are likely to limit its adoption in this particular scenario.

In our cohort, half of our patients have been able to reduce the number of tablets taken daily, resulting in likely cost saving. In addition, when treating a lifelong disorder such as GS, real savings can be made by prescribing and ordering Mg preparations in bulk [3]. However, in the setting of high, long-term requirement for an unpalatable therapy such as Mg supplementation in a small group of individuals, there is an argument for efficacy, tolerability and/or patient preference outweighing cost considerations. Positive patient-reported outcomes in this study suggest that SRMgL is useful in the treatment of chronic hypomagnesaemia and a worthwhile addition to the prescriber's formulary.

## S UPPLEMENTARY DATA

Supplementary data are available online at http://ndt.oxfordjournals.org.

## Supplementary Material

Supplementary Data
