# Supplementary material for: Magnesium lactate in the treatment of Gitelman syndrome: patient-reported outcomes
Source: Nephrol Dial Transplant. 2016 Mar 3;32(3):508–12. doi: 10.1093/ndt/gfw019 (PMC5837242; doi:10.1093/ndt/gfw019)
Supplement: Supplementary Data [file gfw019_supp.pdf]

Patient	Pre-SRMgL form of Mg therapy	Daily no. Mg tabs §		Daily mEq Mg		Serum K (mmol/L)		Serum Mg (mmol/L)	
		Pre-SRMgL	SRMgL	Pre-SRMgL	SRMgL	Pre-SRMgL	SRMgL	Pre-SRMgL	SRMgL
1	Glycerophosphate	4	4	32	28	**3.31**	**3.65**	**0.64**	**0.64**
2	Glycerophosphate	6	4	48	28	2.87	**3.34**	**0.61**	**0.62**
3	Nil	0	2	0	14	**3.35**	**3.33**	**0.62**	**0.65**
4	Chloride/Aspartate	Intolerant	2	0	14	2.58	2.59	**0.77**	**0.75**
5	Glycerophosphate	8	12#	64	84	**3.28**	**3.64**	0.49	0.50
6*	Glycerophosphate	16	8+8	128	120	**4.47**	**4.20**	**0.62**	0.58
7	Chloride	6	6	48	42	2.80	**3.08**	0.53	**0.63**
8	Glycerophosphate	13	10	104	70	**3.56**	**3.81**	0.56	**0.64**
9	Glycerophosphate	12	12	96	84	**3.25**	**3.32**	0.42	0.48
10	Glycerophosphate	6	6	48	42	3.16	**3.36**	**0.78**	**0.70**
11	Oxide/Glycerophosphate	6	4	48	28	**3.55**	**3.62**	**0.66**	**0.68**
12	Glycerophosphate	9	6	72	42	2.75	**3.28**	0.55	**0.64**
13	Glycerophosphate	25	10	200	70	**4.33**	**4.48**	**0.67**	**0.66**
14**	Chloride/Glycinate	6	6	48	42	**4.05**	**3.79**	**0.75**	**0.66**
15	Glycerophosphate	9	6	72	42	2.92	**3.53**	0.55	0.56
16*	Glycerophosphate	32	8+8	256	120	2.92	**3.28**	0.58	0.57
17	Glycerophosphate	4	2	32	14	2.97	**3.97**	**0.65**	**0.65**
18	Glycerophosphate	16	12	128	84	**3.28**	**3.56**	**0.66**	**0.68**
19	Glycerophosphate	N/K	8	N/K	56	**3.20**	3.19	0.51	0.56
20	Glycerophosphate	Intolerant	9	N/K	63	2.89	**3.44**	0.55	**0.60**
21	Nil	0	6	0	42	**3.30**	**3.20**	0.59	**0.68**
22	Glycerophosphate	24	5	192	35	3.11	**3.20**	**0.61**	0.59
23	Chloride/Glycerophosphate	4	4	32	28	**3.45**	**3.58**	**0.61**	**0.67**
24	Nil	0	2	0	14	3.18	N/A	**0.66**	N/A
25	Glycerophosphate	N/K	8	N/K	56	N/A	N/A	N/A	N/A
26	Glycerophosphate	6	Intolerant	48	0	2.93		0.58	
27	Glycerophosphate	4	Intolerant	32	0	**4.01**		**0.67**	
28	Glycerophosphate	6	Intolerant	48	0	**3.39**		**0.67**	

* Patients took 8 SRMgL in combination with 8 Glycerophosphate

** Patient able to discontinue Amiloride

§ Data extracted from most recent medical record

Tried dose up to 18 tabs but diarrhea then led to dose reduction

N/A Insufficient data

N/K Not known

Data in **bold** are at least at our treatment target level
